# A comparison of high-flow nasal cannula and standard facemask as pre-oxygenation technique for general anesthesia: A PRISMA-compliant systemic review and meta-analysis

**DOI:** 10.1097/MD.0000000000028903

**Published:** 2022-03-11

**Authors:** Hsien-Cheng Kuo, Wan-Chi Liu, Chun-Cheng Li, Yih-Giun Cherng, Jui-Tai Chen, Hsiang-Ling Wu, Ying-Hsuan Tai

**Affiliations:** a Department of Anesthesiology, Shuang Ho Hospital, Taipei Medical University, New Taipei City, Taiwan; b Department of Anesthesiology, School of Medicine, College of Medicine, Taipei Medical University, Taipei, Taiwan; c Department of Anesthesiology, Taipei Veterans General Hospital, Taipei, Taiwan; d School of Medicine, National Yang Ming Chiao Tung University, Taipei, Taiwan.

**Keywords:** apneic oxygenation, difficult airway, high-flow nasal oxygenation, transnasal humidified rapid-insufflation ventilatory exchange

## Abstract

**Background:**

Current practice guidelines recommend the use of nasal cannula as an alternative pre-oxygenation method for tracheal intubation. However, the efficacy of high-flow nasal oxygenation versus standard facemask oxygenation has not been fully evaluated.

**Methods:**

We searched PubMed, Cochrane Library, and ClinicalTrials.gov for English-language studies published from January 1, 2000 to November 30, 2021. We included randomized controlled trials which compared high-flow nasal oxygenation and facemask oxygenation as the pre-oxygenation maneuver. Primary outcome was arterial partial pressure of oxygen (PaO_2_) after pre-oxygenation. Secondary outcomes were safe apnea time, arterial desaturation during intubation, lowest peripheral capillary oxygen saturation during intubation, and patient comfort score. Random-effects models and Mantel–Haenszel method were used for data synthesis.

**Results:**

A total of 16 randomized controlled trials and 1148 patients were included. High-flow nasal oxygenation achieved a higher PaO_2_ compared with facemask, mean difference: 64.86 mm Hg (95% confidence interval [CI]: 32.33–97.40, *P* < .0001). Safe apnea time was longer in high-flow nasal oxygenation, mean difference: 131.03 seconds (95% CI: 59.39–202.66, *P* < .0001). There was no difference in the risk of peri-intubation desaturation or lowest peripheral capillary oxygen saturation between groups. Patient comfort score was higher in high-flow nasal oxygenation, mean difference: 1.00 (95% CI: 0.46–1.54, *P* = .0003).

**Conclusion:**

High-flow nasal oxygenation better enhanced PaO_2_ and extended safe apnea time and is not inferior to facemask oxygenation in preventing desaturation during tracheal intubation. High-flow nasal oxygenation may be considered as an alternative method, especially for patients with a potential difficult airway.

## 1 Introduction

Pre-oxygenation before induction of anesthesia is an established procedure to increase the oxygen reservoir in the lungs, to delay the occurrence of desaturation, and to allow more time for laryngoscopy and tracheal intubation.^[1]^ This is particularly important for patients who have poor underlying physiological reserve or undergo rapid sequence induction.^[2]^ Pre-oxygenation is typically performed using a facemask with an adequate seal between the patient and the circuit to deliver oxygen. This standard method has been shown to extend the safe apnea time to 6 minutes for securing the airway in anesthetized patients.^[3]^

Difficult Airway Society 2015 guidelines recommend the use of simple nasal cannula as an alternative method to deliver oxygen continuously during induction of anesthesia.^[4]^ Currently, high flow nasal cannula is widely used to deliver warmed and humidified oxygen flow at a rate over 60 L minute^−1^.^[5]^ Study has shown that high-flow nasal oxygenation (HFNO) generates positive pressure and facilitates carbon dioxide clearance in anesthetized and apneic patients.^[6,7]^ However, it remains uncertain whether HFNO is more efficacious in enhancing arterial oxygenation or reducing peri-intubation desaturation compared with standard facemask oxygenation (FMO). This controversy is potentially due to the study limitations of previous studies, including small sample sizes and discrepancies in oxygenation protocol.^[8–23]^ Recent meta-analyses focused on the efficacy of HFNO in critically ill patients with acute respiratory failure.^[24,25]^ However, these results cannot be generalized to anesthetized patients due to the obvious difference in patients’ health condition and clinical setting.

To better clarify the role of HFNO in pre-oxygenation for general anesthesia (GA), we collected the available published data from randomized controlled trials and conducted this meta-analysis to compare the oxygenation level, safe apnea time, and peri-intubation arterial desaturation between HFNO and FMO as the pre-oxygenation method. Based on previous evidence,^[4–7]^ we hypothesized that HFNO better enhances the level of arterial blood oxygen, prolongs safe apnea time, and reduces the risk of desaturation during intubation in comparison with conventional FMO.

## 2 Methods

### 2.1 Data sources and searches

This meta-analysis used the aggregate data from published studies, did not directly involve human subjects, and therefore did not require the approval of institutional review board. We used the Preferred Reporting Items for Systematic Reviews and Meta-analyses guidelines^[26]^ and performed a comprehensive search using PubMed, Cochrane Library, and ClinicalTrials.gov for published studies in English from January 1, 2000 to November 30, 2021. We searched with the keywords “high-flow nasal oxygen,” “high-flow nasal oxygenation,” “high-flow nasal cannula,” “transnasal humidified rapid-insufflation ventilatory exchange,” “pre-oxygenation,” “apneic oxygenation,” and “OptiFlow.” References of articles and relevant meta-analyses were also reviewed to confirm that no study was missed.

### 2.2 Eligibility criteria and study quality assessment

Included studies met all the following criteria: subjects ≥18 years of age, uses of HFNO for pre-oxygenation, comparisons of high-flow nasal cannula and facemask, reporting outcomes of arterial partial pressure of oxygen (PaO_2_), safe apnea time, oxygen desaturation during intubation, lowest peripheral capillary oxygen saturation (SpO_2_) during intubation or patient comfort score, and articles published in peer-reviewed journals. We excluded observational studies, review articles or editorials, studies evaluating critically ill patients with respiratory failure, and studies not reporting outcomes of interest. Two authors (HCK and WCL) independently reviewed and collected data, including study design, characteristics of subjects, and study outcomes. The third blinded reviewer (YGC) resolved any disagreements between reviewers. The quality of randomized controlled trials was appraised by HCK and WCL using the Cochrane Handbook for Systematic Reviews of Interventions.^[27]^ Any disagreement was resolved via group discussions.

### 2.3 Outcome measurement

Patients were classified into the HFNO group if they underwent pre-oxygenation using a high-flow nasal cannula with a flow of 100% oxygen >30 L minute^−1^. Patients were classified into the FMO group if pre-oxygenation maneuver was performed using a standard anesthetic facemask with 100% oxygen. Primary outcome was the level of PaO_2_ after pre-oxygenation. Secondary outcomes were safe apnea time, which was defined as the interval between start of apnea verified by capnography and SpO_2_ reaching 90% to 95% or endotracheal tube in place,^[10–12,16,18,20–22]^ arterial desaturation (SpO_2_ below 90–95%) during intubation, lowest SpO_2_ during intubation, and patient comfort score. Patient comfort score was graded by the numeric rating scale, ranging from 0 to 10. The ascertainment of safe apnea time and arterial desaturation were based on the definitions described in the primary studies.

### 2.4 Data synthesis and analysis

Data synthesis was performed using random-effects models and Mantel–Haenszel method to generate mean difference and risk ratio (RR) by using RevMan software, version 5.3 (Nordic Cochrane Centre, Cochrane Collaboration, Copenhagen, Denmark). The *I*^2^ statistics were used to check for quantitative heterogeneity of results^[28]^; it defines low heterogeneity with *I*^2^ < 25%, moderate heterogeneity with *I*^2^ between 25% and 50%, and high heterogeneity with *I*^2^ >50%. A funnel plot was used to assess a potential publication bias. Visual estimation was performed to examine the asymmetry of the funnel plot. We estimated and obtained the sample mean and standard deviation from the sample size, median, interquartile range, and/or range of the primary studies using Wan et al's method.^[29]^ Subgroup analyses by obese and non-obese people were conducted to compare the efficacy of HFNO and FMO in the 2 populations. As a sensitivity test, we excluded the studies of healthy volunteers (Pillai et al^[9]^ and Hanouz et al^[14]^) to exclusively examine the efficacy of HFNO and FMO in surgical and anesthetized patients. A 2-sided significance level of 0.05 was used to assess statistically significant difference.

## 3 Results

**Figure 1:**
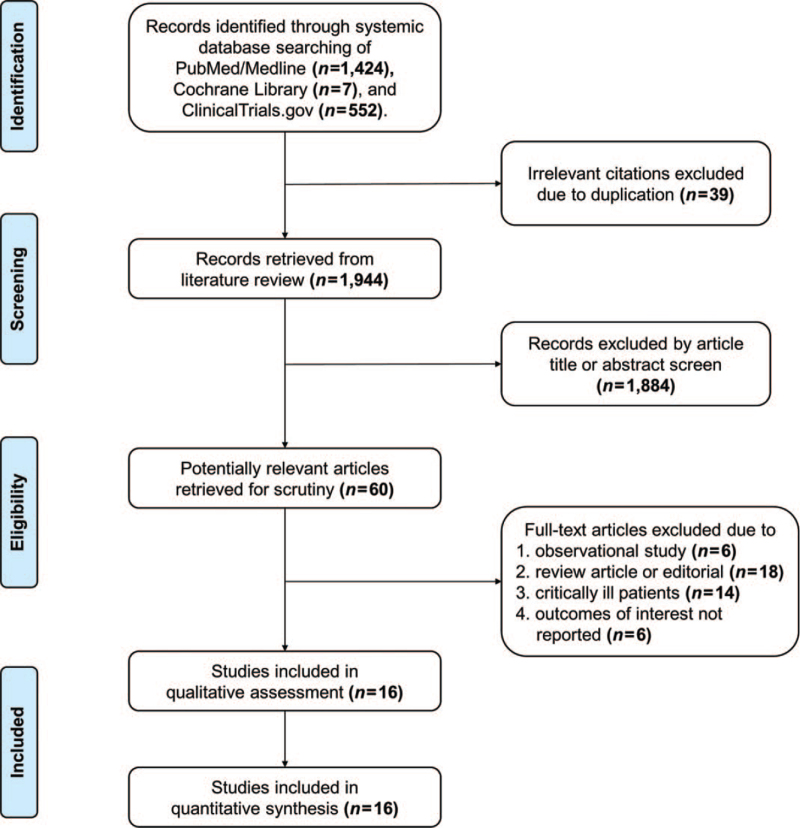
Flow diagram of the review process according to the Preferred Reporting Items for Systematic Reviews and Meta-analyses (PRISMA) statement.

The flow diagram of Preferred Reporting Items for Systematic Reviews and Meta-analyses is described in Fig. 1. The systemic review identified a total of 16 randomized controlled trials,^[8–23]^ which enrolled 1148 patients. Patients were divided into the HFNO group (n = 576) and FMO group (n = 572). Baseline characteristics of the included studies are listed in Table 1. Indications for pre-oxygenation were reported in all studies, including induction of anesthesia^[8,10–13,15–23]^ and healthy volunteers.^[9,14]^ Four studies evaluated obese patients for surgery.^[8,15,16,22]^ Two studies recruited healthy pregnant women who required tracheal intubation for elective cesarean section.^[20,23]^

**Table 1:** Characteristics of the included randomized controlled trials.

Author	Country	Characteristics of subjects	No. of subjects	Protocol of HFNO	Protocol of FMO	Duration of pre-oxygenation	Apneic oxygenation in HFNO	Apneic oxygenation in FMO	Neuro-muscular blocker	Patient position
Heinrich et al, 2014^[8]^	Germany	Morbidly obese patients undergoing RSI for laparoscopic bariatric surgery	HFNO: 11 FMO: 11	OptiFlow using a flow of 100% oxygen 50 L min^−1^ with mouth closed	Spontaneous breathing with 100% oxygen flow of 12 L min^−1^	7 min	OptiFlow using a flow of 100% oxygen 50 L min^−1^	100% oxygen flow of 12 L min^−1^ using a facemask	Succinylcholine	30° head up
Pillai et al, 2016^[9]^	United Kingdom	Healthy volunteers	HFNO: 10 FMO: 10	OptiFlow using a flow of 100% oxygen 60 L min^−1^ with mouth closed	Spontaneous breathing with 100% oxygen flow of 10 L min^−1^	3 min	NA	NA	NA	45° head up
Mir et al, 2017^[10]^	United Kingdom	Patients requiring RSI for emergency surgery	HFNO: 20 FMO: 20	OptiFlow using a flow of 100% oxygen 30–70 L min^−1^	Spontaneous breathing with 100% oxygen flow of 12 L min^−1^	3 min	OptiFlow using a flow of 100% oxygen 70 L min^−1^	100% oxygen flow of 12 L min^−1^ using a facemask	Rocuronium 1 mg kg^−1^	Not reported
Rajan et al, 2018^[11]^	India	Patients for direct laryngoscopy under GA without tracheal intubation	HFNO: 5 FMO: 5	OptiFlow using a flow of 100% oxygen 30 L min^−1^	Spontaneous breathing with 100% oxygen flow of 6 L min^−1^	3 min	OptiFlow sing a flow of 100% oxygen 60 L min^−1^	Oxygen flow at 12 L min^−1^ using a nasopharyngeal catheter	Atracurium 0.5 mg kg^−1^	NR
Lodenius et al, 2018^[12]^	Sweden	Patients requiring RSI for emergency surgery	HFNO: 40 FMO: 39	OptiFlow using a flow of 100% oxygen 40 L min^−1^	Spontaneous breathing with 100% oxygen flow of 10 L min^−1^	3 min	OptiFlow using a flow of 100% oxygen 70 L min^−1^	100% oxygen flow of 10 L min^−1^ using a facemask	Succinylcholine 1.2 mg kg^−1^ or rocuronium 0.9 mg kg^−1^	25° head up
Ng et al, 2018^[13]^	Australia	Patients requiring intubation for neurosurgical operation	HFNO: 24 FMO: 24	OptiFlow using a flow of 100% oxygen 30–50 L min^−1^	Spontaneous breathing with 100% oxygen flow of 10 L min^−1^	5 min	OptiFlow using a flow of 100% oxygen 50 L min^−1^	Bag-mask ventilation to keep EtCO_2_ between 35 and 40 mm Hg	Rocuronium 1.0 mg kg^−1^	Sniffing position
Hanouz et al, 2019^[14]^	France	Healthy volunteers	HFNO: 50 FMO: 50	OptiFlow using a flow of 100% oxygen 60 L min^−1^ with mouth closed	Spontaneous breathing with 100% oxygen flow of 12 L min^−1^	3 min	NA	NA	NA	NR
Vourc’h et al, 2019^[15]^	France	Obese patients requiring RSI for elective surgery	HFNO: 50 FMO: 50	OptiFlow using a flow of 100% oxygen 60 L min^−1^	Pressure support mode with a 10 cm H_2_O pressure support, 100% oxygen	4 min	OptiFlow using a flow of 100% oxygen 60 L min^−1^	Pressure support mode with a 10 cm H_2_O pressure support, 100% oxygen	Succinylcholine, rocuronium, or none	Ramped position
Wong et al, 2019^[16]^	Canada	Obese patients requiring intubation for elective surgery	HFNO: 20 FMO: 20	OptiFlow using a flow of 100% oxygen 40 L min^−1^	Spontaneous breathing with 100% oxygen flow of 15 L min^−1^	3 min	OptiFlow using a flow of 100% oxygen 60 L min^−1^	100% oxygen flow of 15 L min^−1^ using a facemask	Rocuronium 0.6 mg kg^−1^	30° head up
Tremey et al, 2020^[17]^	France	Patients requiring intubation for an elective surgical procedure	HFNO: 30 FMO: 31	OptiFlow using a flow of 100% oxygen 30 L min^−1^ with mouth closed	Spontaneous breathing with 100% oxygen	≥3 min	OptiFlow using a flow of 100% oxygen 60 l min^−1^	100% oxygen using a facemask	Rocuronium 0.6 mg kg^−1^	Sniffing position for FMO; semi-sitting position (40°) for HFNO
Hua et al, 2020^[18]^	China	Elderly patients requiring intubation or laryngeal mask for general anesthesia	HFNO: 30 FMO: 28	OptiFlow using a flow of 100% oxygen 30 L min^−1^	Spontaneous breathing with 100% oxygen flow of 10 L min^−1^	5 min	OptiFlow using a flow of 100% oxygen 70 L min^−1^	100% oxygen flow of 10 L min^−1^ using a facemask	Cisatracurium 0.1–0.2 mg kg^−1^	Supine position
Sjöblom et al, 2021^[19]^	Sweden and Switzerland	Patients requiring RSI for emergency surgery	HFNO: 174 FMO: 175	OptiFlow using a flow of 100% oxygen 30–50 L min^−1^	Spontaneous breathing with 100% oxygen flow of 10 L min^−1^	≥3 min	OptiFlow using a flow of 100% oxygen 70 L min^−1^	100% oxygen flow of 10 L min^−1^ using a facemask	Succinylcholine, rocuronium, or combination	Reverse trendelenburg position
Osman et al, 2021^[20]^	Egypt	Healthy parturients requiring intubation for elective cesarean section	HFNO: 50 FMO: 50	OptiFlow using a flow of 100% oxygen 30–50 L min^−1^	Spontaneous breathing with 100% oxygen flow of 6 L min^−1^	3 min	OptiFlow using a flow of 100% oxygen 50 L min^−1^	Simple nasal cannula with 100% oxygen flow of 6 L min^−1^	Atracurium 0.5 mg kg^−1^	Ramped position with left lateral tilt
Lyons et al, 2021^[21]^	Ireland	Patients requiring intubation for an elective surgical procedure	HFNO: 25 FMO: 26	OptiFlow using a flow of 100% oxygen 50 L min^−1^	Spontaneous breathing with 100% oxygen flow of 15 L min^−1^	≥3 min	OptiFlow using a flow of 100% oxygen 50 L min^−1^	100% oxygen flow of 15 L min^−1^ using a facemask	Rocuronium 1.0 mg kg^−1^	NR
Rosén et al, 2021^[22]^	Sweden	Obese patients undergoing intubation for laparoscopic bariatric surgery	HFNO: 20 FMO: 16	OptiFlow using a flow of 100% oxygen 70 L min^−1^ with mouth closed	Spontaneous breathing with 100% oxygen flow of 8 L min^−1^ with a PEEP of 7 cm H2O	5 min	OptiFlow using a flow of 100% oxygen 70 L min^−1^	Bag-mask ventilation with 100% oxygen	Rocuronium 0.6 mg kg^−1^ lean body weight	Ramped sniffing position
Zhou et al, 2021^[23]^	China	Healthy parturients requiring RSI for cesarean section	HFNO: 17 FMO: 17	OptiFlow using a flow of 100% oxygen 50 L min^−1^	Spontaneous breathing with 100% oxygen flow of 10 L min^−1^	3 min	OptiFlow using a flow of 100% oxygen 50 L min^−1^	100% oxygen flow of 10 L min^−1^ using a facemask	Rocuronium	NR

EtCO_2_ = end-tidal carbon dioxide, FMO = facemask oxygenation, GA = general anesthesia, HFNO = high-flow nasal oxygenation, NA = not applicable, NR = not reported, PEEP = positive end-expiratory pressure, RSI = rapid sequence induction.

All studies used OptiFlow system (AIRVO 2; Fisher and Paykel Healthcare Ltd., New Zealand) for HFNO.^[8–23]^ HFNO was performed with a warmed and humidified flow of 100% oxygen with a rate of 30 to 70 L minute^−1^ during pre-oxygenation. The nasal cannula flow was escalated to 50 to 70 L minute^−1^ for apneic oxygenation in anesthetized patients.^[8,10–13,15–23]^ Lyons et al^[21]^ randomized patients into 3 groups, facemask, high-flow nasal cannula only, or high-flow nasal cannula plus a mouthpiece. We used the data of high-flow nasal cannula only for meta-analysis to reduce the heterogeneity of oxygenation method.^[21]^

For FMO, 14 studies used a well-sealed facemask connected to an anesthetic circuit and allowed patients to breath spontaneously with 100% oxygen flow of 6 to 15 L minute^−1^.^[8–14,16–21,23]^ One study performed FMO by using ventilators with a 10 cm H_2_O pressure support and 100% oxygen.^[15]^ One study used positive end-expiratory pressure (PEEP) of 7 cm H_2_O for FMO.^[22]^ Heinrich et al^[8]^ divided participants into 3 groups, facemask without positive pressure, facemask with positive pressure, or high-flow nasal cannula. We used the data of facemask without positive pressure for meta-analysis.^[8]^ Duration of pre-oxygenation ranged from 3 to 7 minutes.

After apnea occurred, one study used a nasopharyngeal catheter to deliver oxygen at a flow of 12 L minute^−1^.^[11]^ One study used a simple nasal cannula with 100% oxygen flow of 6 L minute^−1^.^[20]^ Two studies used bag-mask ventilation.^[13,22]^ Other studies used a facemask to deliver 100% oxygen flow of 10 to 15 L minute^−1^.^[8,10,12,15–19,21,23]^ Heinrich et al^[8]^ measured the PaO_2_ at 1, 3, 5, 7, and 8.5 minutes during pre-oxygenation, and we used the data of 3 minutes. Rosén et al^[22]^ measured the PaO_2_ at 2.5 and 5.0 minutes during pre-oxygenation, and we used the data of 2.5 minutes.

### 3.1 Study quality assessment

Table 2 shows the assessment of study quality: 14 studies conducted a high-quality randomization with allocation concealment.^[9–11,13–23]^ All trials were open-label due to the different appearance of the oxygenation devices and difficulty in blinding participants and anesthetists.

**Table 2:** Study quality assessment of randomized controlled trials.

Author	Random allocation	Allocation concealment	Blinding	Any loss to follow-up	Analysis
Heinrich et al, 2014^[8]^	PY	NR	Patients: CN; caregivers: CN; data collectors: NR; adjudicators: NR; data analysis: NR.	CN	ITT: PY for efficacy outcomes Data for primary efficacy assessment available for 100% of randomized patients
Pillai et al, 2016^[9]^	DY	PY	Patients: CN; caregivers: CN; data collectors: NR; adjudicators: NR; data analysis: NR.	CN	ITT: DY for efficacy outcomes Data for primary efficacy assessment available for 100% of randomized patients
Mir et al, 2017^[10]^	DY	DY	Patients: CN; caregivers: CN; data collectors: NR; adjudicators: NR; data analysis: NR.	CN	ITT: DY for efficacy outcomes Data for primary efficacy assessment available for 100% of randomized patients
Rajan et al, 2018^[11]^	DY	DY	Patients: CN; caregivers: CN; data collectors: NR; adjudicators: NR; data analysis: NR.	CN	ITT: DY for efficacy outcomes Data for primary efficacy assessment available for 100% of randomized patients
Lodenius et al, 2018^[12]^	PY	CN	Patients: CN; caregivers: CN; data collectors: NR; adjudicators: NR; data analysis: NR.	CN	ITT: PN for efficacy outcomes Data for primary efficacy assessment available for 98.8% of randomized patients
Ng et al, 2018^[13]^	DY	DY	Patients: CN; caregivers: CN; data collectors: CN; adjudicators: NR; data analysis: NR.	CN	ITT: PN for efficacy outcomes Data for primary efficacy assessment available for 96.0% of randomized patients
Hanouz et al, 2019^[14]^	PY	PY	Patients: CN; caregivers: CN; data collectors: NR; adjudicators: NR; data analysis: NR.	CN	ITT: DY for efficacy outcomes Data for primary efficacy assessment available for 100% of randomized patients
Vourc’h et al, 2019^[15]^	DY	DY	Patients: CN; caregivers: CN; data collectors: NR; adjudicators: NR; data analysis: NR.	CN	ITT: DY for efficacy outcomes Data for primary efficacy assessment available for 100% of randomized patients
Wong et al, 2019^[16]^	DY	DY	Patients: CN; caregivers: CN; data collectors: NR; adjudicators: NR; data analysis: NR.	CN	ITT: DY for efficacy outcomes Data for primary efficacy assessment available for 100% of randomized patients
Tremey et al, 2020^[17]^	DY	PY	Patients: CN; caregivers: CN; data collectors: NR; adjudicators: NR; data analysis: NR.	CN	ITT: DY for efficacy outcomes Data for primary efficacy assessment available for 98.4% of randomized patients
Hua et al, 2020^[18]^	DY	PY	Patients: CN; caregivers: CN; data collectors: NR; adjudicators: NR; data analysis: NR.	CN	ITT: PN for efficacy outcomes Data for primary efficacy assessment available for 98.3% of randomized patients
Sjöblom et al, 2021^[19]^	DY	DY	Patients: CN; caregivers: CN; data collectors: NR; adjudicators: NR; data analysis: NR.	CN	ITT: PN for efficacy outcomes Data for primary efficacy assessment available for 99.7% of randomized patients
Osman et al, 2021^[20]^	DY	DY	Patients: CN; caregivers: CN; data collectors: NR; adjudicators: NR; data analysis: NR.	CN	ITT: PN for efficacy outcomes Data for primary efficacy assessment available for 94.3% of randomized patients
Lyons et al, 2021^[21]^	DY	DY	Patients: CN; caregivers: CN; data collectors: NR; adjudicators: NR; data analysis: NR.	CN	ITT: PN for efficacy outcomes Data for primary efficacy assessment available for 91.1% of randomized patients
Rosén et al, 2021^[22]^	DY	DY	Patients: CN; caregivers: CN; data collectors: NR; adjudicators: NR; data analysis: NR.	CN	ITT: PN for efficacy outcomes Data for primary efficacy assessment available for 90.0% of randomized patients
Zhou et al, 2021^[23]^	DY	DY	Patients: CN; caregivers: CN; data collectors: NR; adjudicators: NR; data analysis: NR.	CN	ITT: PN for efficacy outcomes Data for primary efficacy assessment available for 85.0% of randomized patients

CN = certainly no, DY = definitely yes, ITT = intention to treat, NR = not reported, PN = probably no, PY = probably yes.

### 3.2 PaO_2_ after pre-oxygenation

**Figure 2:**
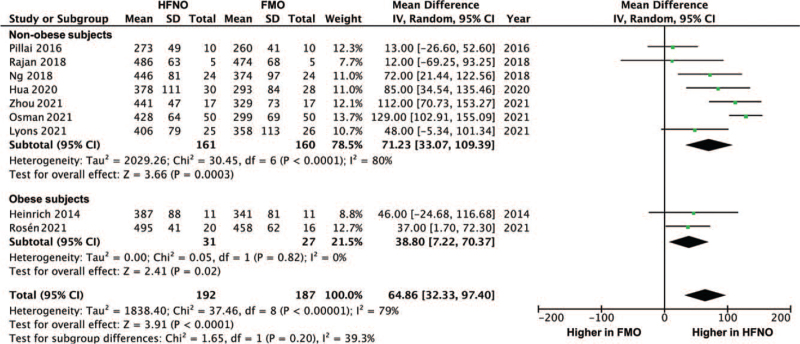
Forest plot of arterial partial pressure of oxygen (mm Hg) after pre-oxygenation between HFNO and FMO groups. CI = confidence interval, FMO = facemask oxygenation, HFNO = high-flow nasal oxygenation.

When data were pooled across studies, the analysis showed that HFNO achieved a higher PaO_2_ after pre-oxygenation compared with FMO, mean difference: 64.86 mm Hg (95% confidence interval [CI]: 32.33–97.40, *P* < .0001; *I*^2^ = 79%) (Fig. 2). The difference was significant both in obese subjects (38.80, 95% CI: 7.22–70.37, *P* = .02; *I*^2^ = 0%) and non-obese subjects (71.23, 95% CI: 33.07–109.39, *P* = .0003; *I*^2^ = 80%). Funnel plot revealed no obvious publication bias. The difference in the PaO_2_ between HFNO and FMO in surgical patients is shown in the Figure S1, Supplemental Digital Content.

### 3.3 Safe apnea time

**Figure 3:**
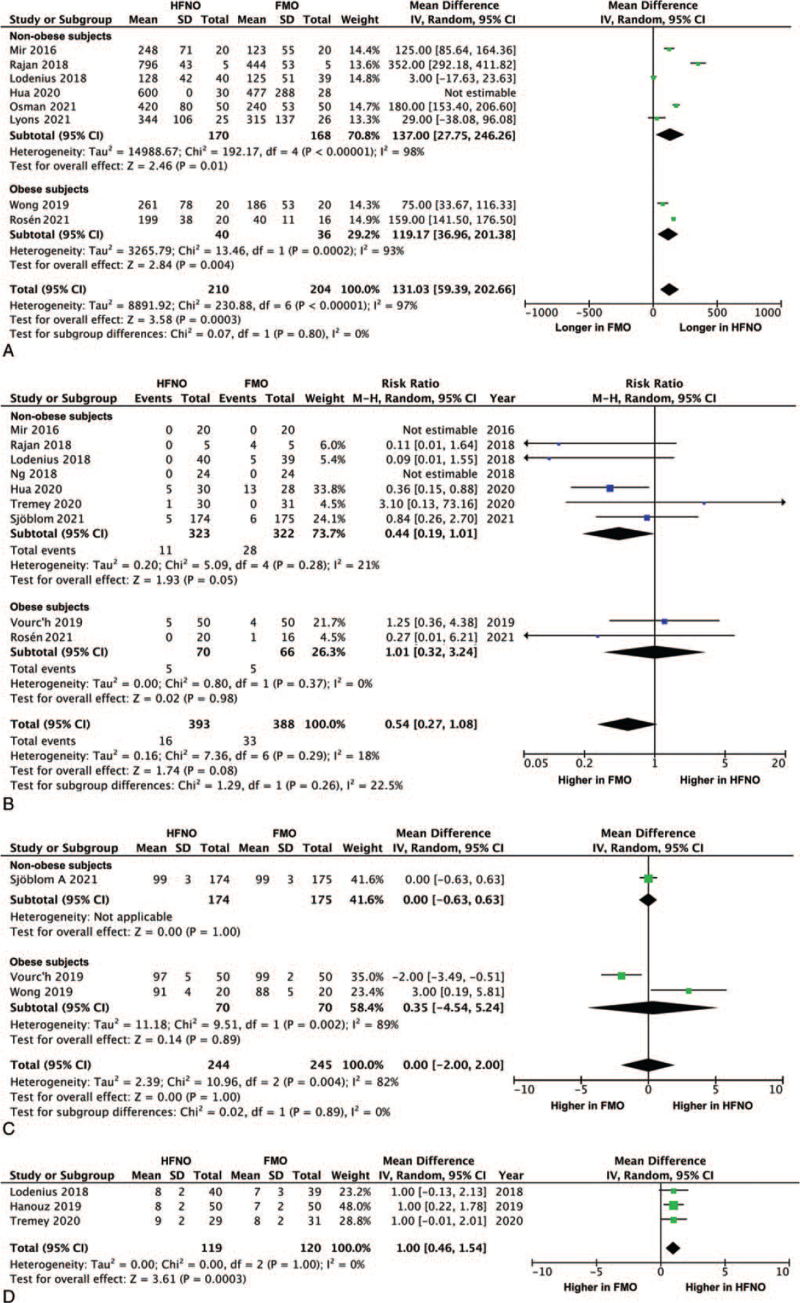
Forest plot of (A) safe apnea time (second), (B) arterial desaturation during intubation, (C) lowest peripheral capillary oxygen saturation during intubation (%), and (D) patient comfort score (numeric rating scale, 0–10) between HFNO and FMO groups. CI = confidence interval, FMO = facemask oxygenation, HFNO = high-flow nasal oxygenation, M–H = Mantel–Haenszel.

Safe apnea time was longer in patients using HFNO compared with FMO, mean difference: 131.03 seconds (95% CI: 59.39–202.66, *P* < .0001; *I*^2^ = 97%) (Fig. 3A). The safe apnea time was significantly greater in HFNO compared with FMO both in obese patients (119.17, 95% CI: 36.96–201.38, *P* = .004; *I*^2^ = 93%) and non-obese patients (137.00, 95% CI: 27.75–246.26, *P* = .01; *I*^2^ = 98%). Funnel plot revealed no obvious publication bias.

### 3.4 Arterial desaturation during intubation

There was no difference in the risk of arterial desaturation during intubation between HFNO and FMO in all studies (RR: 0.54, 95% CI: 0.27–1.08, *P* = .08; *I*^2^ = 18%) (Fig. 3B). However, there was a trend towards a lower risk of desaturation in the non-obese patients (RR: 0.44, 95% CI: 0.19–1.01, *P* = .05; *I*^2^ = 21%). Funnel plot revealed no obvious publication bias.

### 3.5 Lowest SpO_2_ during intubation

There was no difference in the lowest SpO_2_ during intubation between 2 groups, mean difference: 0% (95% CI: –2.00–2.00, *P* = 1.00; *I*^2^ = 82%) (Fig. 3C).

### 3.6 Patient comfort score

Patients undergoing HFNO had a higher comfort score compared with FMO, mean difference: 1.00 (95% CI: 0.46–1.54, *P* = .0003; *I*^2^ = 0%) (Fig. 3D). The results of sensitivity test are shown in the Figure S2, Supplemental Digital Content.

## 4 Discussion

This study was the first meta-analysis of randomized controlled trials to specifically compare the efficacy of high-flow nasal cannula and standard facemask in pre-oxygenation for anesthetized patients. Our analyses showed that HFNO achieved a higher PaO_2_ after pre-oxygenation, extended safe apnea time, and improved patient comfort compared with FMO. The risk of desaturation and lowest SpO_2_ during intubation were similar between 2 techniques. These results suggest that HFNO appears to be a practicable method for pre-oxygenation in the setting of GA.

Difficult Airway Society 2015 guidelines recommend the use of nasal cannula in pre-oxygenation,^[4]^ which is primarily based on the studies before 2010 (with nasal insufflation of oxygen at a flow of only 5 L minute^−1^)^[30,31]^ or observational studies.^[32]^ In the recent decade, randomized controlled trials have shown that transnasal humidified high-flow oxygen with a rate up to 70 L minute^−1^ prolongs safe apnea time during tracheal intubation for severely hypoxemic patients.^[24,25,32,33]^ Although guidelines recommend the use of nasal cannula in patients at high risk of difficult airway, it's efficacy as a pre-oxygenation method has not been thoroughly evaluated.^[4]^ Our study provided the evidence that HFNO with apneic oxygenation enhances PaO_2_ better and is not inferior to FMO in preventing hypoxia during tracheal intubation. For patients with an anticipated difficult airway, studies have demonstrated that high-flow nasal oxygen therapy improved oxygen saturation and reduced the risk of desaturation during awake fiberoptic tracheal intubation compared with facemask ventilation.^[34,35]^ These results suggest that HFNO may serve as an ideal pre-oxygenation technique for performing potentially difficult intubations.

There are 2 physiological mechanisms underlying the benefits of high-flow nasal oxygen therapy in oxygenation and gaseous exchange. First, HFNO generates a low level of positive pressure, mean 2.7 cm H_2_O at a gas flow rate of 35 L minute^−1^ in healthy adults.^[6]^ HFNO for apneic oxygenation extends safe apnea time compared with conventional oxygen therapy.^[4,7,10,11,16,34,36]^ Second, transnasal humidified rapid-insufflation ventilatory exchange in HFNO may facilitate gaseous exchange and enhance carbon dioxide clearance through the interaction between cardiogenic oscillations and supraglottic flow vortices created by nasal gas flow.^[7,36]^ By contrast, a recent randomized trial refuted the advantage of HFNO over spontaneous ventilation in carbon dioxide washout among adults undergoing microlaryngoscopy.^[37]^ More studies are needed to clarify the efficacy of HFNO in improving gaseous exchange during apnea.

Patients with obesity have reduced respiratory reserve due to their lower vital capacity, functional residual capacity, and lung compliance.^[38]^ The apnea time of SpO_2_ dropping to 90% after FMO is <3 minutes in obese patients compared with 6 minutes in the non-obese.^[39]^ The greater risk of oxygen desaturation in obese patients warrants further development of pre-oxygenation techniques for anesthesia care. However, it remains inconclusive whether HFNO is superior to FMO in preventing perioperative desaturation in obese patients.^[8,15,16,22,40–42]^ Heinrich et al^[8]^ reported that HFNO for 3 minutes generated the highest PaO_2_ compared with oxygen insufflation or continuous positive airway pressure via a facemask. Similarly, HFNO prolonged the safe apnea time to SpO_2_ 95% by 76 seconds and enhanced minimum SpO_2_ in morbidly obese patients.^[16]^ On the contrary, Vourc’h et al^[15]^ showed that HFNO produced a lower end-tidal oxygen level after tracheal intubation and carried a higher risk of desaturation. Rosén et al^[22]^ showed that FMO was superior to HFNO in enhancing end-tidal fraction of oxygen. The use of HFNO after tracheal extubation was demonstrated to prevent hypoxemia among obese patients following bariatric surgery in one study^[40]^ but not another.^[41]^ In propofol sedation for colonoscopy, the desaturation rate was similar between HFNO and standard nasal cannula.^[42]^ These disagreements may come from the discrepancies in the oxygenation protocol and study outcomes. Vourc’h et al^[15]^ used pressure support and Rosén et al^[22]^ used PEEP for FMO. Furthermore, the inconsistent use of neuromuscular blocking drugs in the study of Vourc’h et al^[15]^ might also affect the efficacy of HFNO. The use of neuromuscular blocking agents is associated with a higher success rate of tracheal intubation.^[43]^ Intubation without neuromuscular blockade may increase the risk of oxygen desaturation, which potentially underestimates the potential benefit of HFNO in preventing desaturation. More studies are warranted to elucidate the optimal strategy of applying HFNO to peri-procedural oxygenation for obese patients.

A simple facemask typically provides supplemental oxygen with a flow rate of 5 to 10 L minute^−1^ and a fraction of inspired oxygen of 0.35 to 0.55.^[44]^ The oxygen flow rate >10 L minute^−1^ cannot further increase the fraction of inspired oxygen in a simple facemask.^[44]^ In addition, the facemask is necessarily removed during tracheal intubation and therefore cannot deliver an oxygen flow regardless of the flow rate. Based on our analytical results, we reason that FMO with an oxygen flow >10 L minute^−1^ can hardly outperform HFNO in terms of PaO_2_ and safe apnea time. More studies are needed to evaluate the efficacy of HFNO and FMO with varying oxygen flow rates, particularly for patients with poor respiratory reserve.

Attention to some limitations of this study is needed. First, the number of subjects enrolled in the meta-analysis was only modest, and some subgroup analyses may have inadequate statistical power. Second, the outcomes of interest are not available for all included studies. Third, the heterogeneity of results was high in some analyses, which may relate to the variations in characteristic of subjects (healthy volunteers or patients), protocol of oxygenation (oxygen flow rate, use of pressure support or PEEP, and use of neuromuscular blocking agents or not), and definitions of outcomes. Fourth, given that the individual date of included clinical trials are unavailable, we could not compare the efficacy of HFNO and FMO in some subgroups, such as men or women and people with different body mass indexes.

In conclusion, HFNO for pre-oxygenation achieved a higher PaO_2_, extended safe apnea time, and improved patient comfort compared with standard FMO. The risk of desaturation and lowest SpO_2_ during intubation were similar between 2 methods. These results suggest that HFNO appears to be an ideal and useful technique for pre-oxygenation in the setting of GA. HFNO may be considered as an alternative to standard FMO, especially for patients at risk of difficult intubation.

## Author contributions

**Conceptualization:** Chun-Cheng Li, Ying-Husan Tai.

**Data curation:** Hsien-Cheng Kuo, Wan-Chi Liu.

**Formal analysis:** Hsien-Cheng Kuo.

**Funding acquisition:** Ying-Husan Tai.

**Investigation:** Ying-Husan Tai.

**Resources:** Yih-Giun Cherng.

**Supervision:** Ying-Husan Tai.

**Validation:** Yih-Giun Cherng.

**Writing – original draft:** Hsien-Cheng Kuo, Wan-Chi Liu.

**Writing – review & editing:** Chun-Cheng Li, Jui-Tai Chen, Hsiang-Ling Wu, Ying-Husan Tai.

## Supplementary Material

Supplemental Digital Content

## Supplementary Material

Supplemental Digital Content
