# Supplementary figures and images for: A comparison of high-flow nasal cannula and standard facemask as pre-oxygenation technique for general anesthesia: A PRISMA-compliant systemic review and meta-analysis
Source: Medicine (Baltimore). 2022 Mar 11;101(10):e28903. doi: 10.1097/MD.0000000000028903 (PMC8913129; doi:10.1097/MD.0000000000028903)

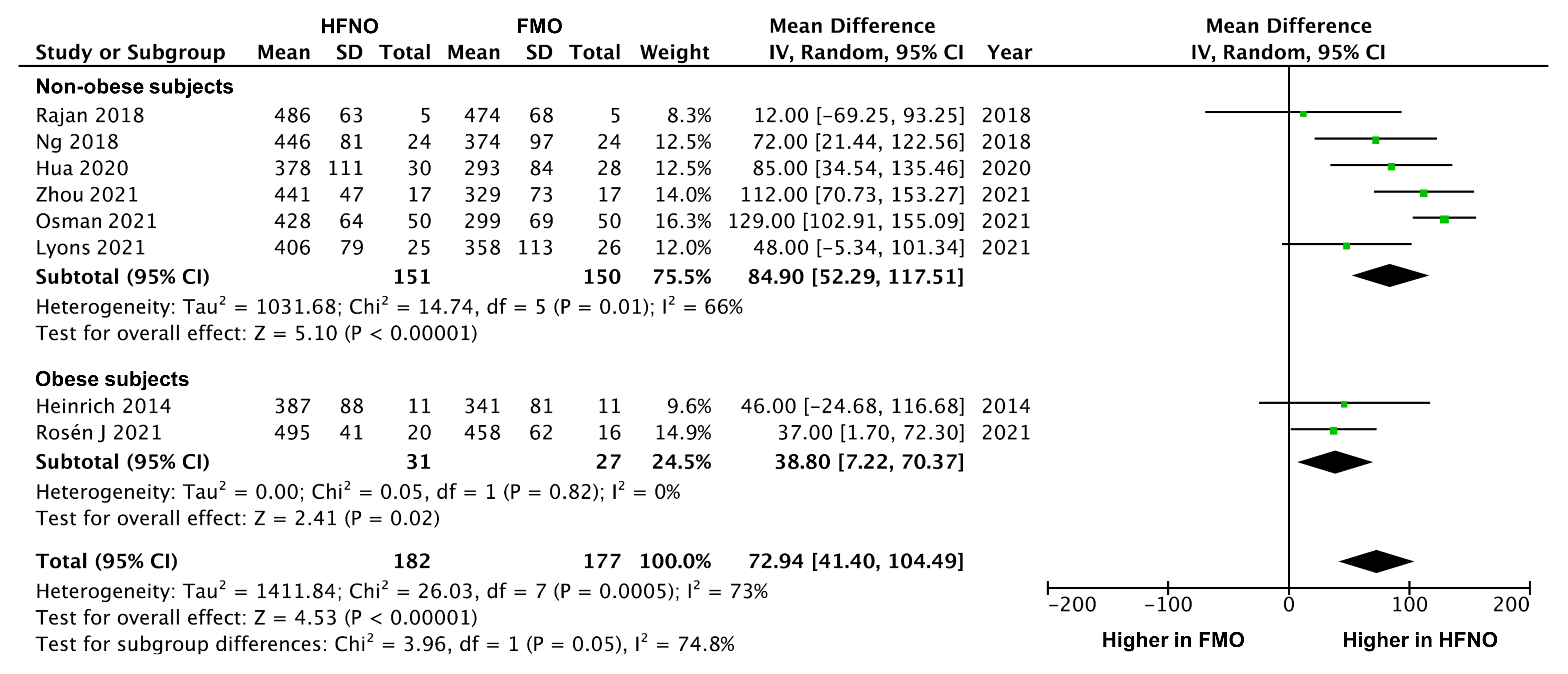

Supplement: Supplemental Digital Content [file medi-101-e28903-s001.tif]

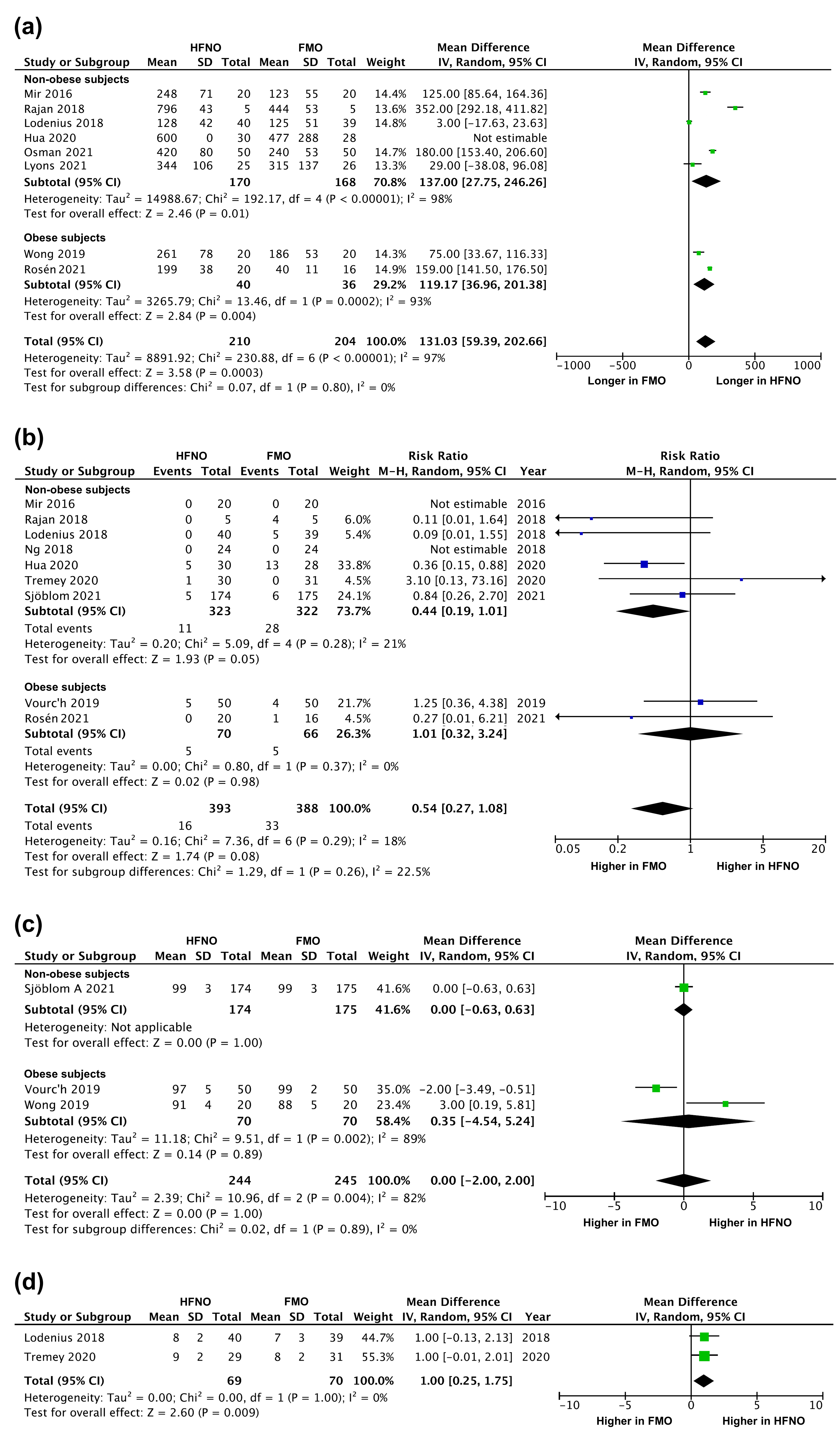

Supplement: Supplemental Digital Content [file medi-101-e28903-s002.tif]
